# Effects of Forest Environments in Attenuating D-Galactose-Induced Immunosenescence: Insights from a Murine Model

**DOI:** 10.3390/biology14080998

**Published:** 2025-08-05

**Authors:** Yanling Li, Xiaocong Li

**Affiliations:** 1 School of Aeronautics and Astronautics, Southwest University of Science and Technology, Mianyang 621010, China; lllyanling@swust.edu.cn; 2 School of Civil Engineering and Architecture, Southwest University of Science and Technology, Mianyang 621010, China; 3 College of Life Science and Agri-Forestry, Southwest University of Science and Technology, Mianyang 621010, China

**Keywords:** urban forests, immunosenescence, healthy aging, D-galactose model, environmental immunomodulation

## Abstract

With the global aging population on the rise, identifying environmental factors that modulate immunosenescence is critical for health interventions. While urban green spaces are known to confer health benefits, the long-term effects of forest exposure on immunosenescence remain unclear. This study investigated the differential impacts of urban forest versus urban environments on immunosenescence using a D-galactose-induced murine model. Mice were assigned to urban or forest environments for 8 weeks, with serum cytokines (IL-2, IL-6, TNF-α, IFN-γ), T-cell subsets, and organ indices analyzed. Forest environments exhibited significantly higher humidity and negative air ion concentrations alongside lower noise levels compared to urban settings. Aged forest-exposed mice showed attenuated immunosenescence markers, including significantly lower IL-6 levels (*p* < 0.01) and improved thymic indices, suggesting urban forest environments may mitigate immune decline. These findings highlight the potential of urban forests in promoting healthy aging, advocating for their integration into urban planning. Further human studies are warranted to translate these findings into public health strategies.

## Simple Summary

With the intensification of global population aging, exploring the influence of living environments on immunosenescence has become a crucial interdisciplinary health topic. This study used a mouse model to compare the effects of urban forests and typical urban environments on immune aging. The results revealed that mice chronically exposed to forest environments exhibited significantly better immune profiles than those in urban settings, characterized by lower levels of inflammation and healthier immune organs. Factors such as better air quality, lower noise levels, and higher humidity in forest environments may contribute to these positive effects. The findings suggest that urban forest green spaces may help slow immune system decline and promote healthy aging. This study provides biological evidence that may inform future urban planning and public health policies.

## 1. Introduction

With ongoing urban development, the spatial structure of cities is undergoing continuous transformation, and in some regions, urban green space development is poorly coordinated with building expansion [1]. Concurrently, population aging is accelerating, intensifying the burden on public health. By 2059, it is projected that two-thirds of the global population will reside in urban areas [2], leading to reduced accessibility to green spaces and increasing health risks associated with urban air pollution and noise exposure. For older adults, these environmental risks are further exacerbated by immunosenescence, an age-associated decline in immune function, which contributes to increased disease susceptibility and reduced vaccine efficacy in this population [3]. Environmental interventions to regulate immunosenescence thus represent a promising non-pharmacological approach to health promotion.

In recent years, nature-based interventions such as forest therapy have garnered growing attention for their potential health benefits. Accumulating evidence suggests that the unique microclimatic characteristics of forest environments may help mitigate the adverse effects of urban stressors. Compared to built environments, forests typically exhibit higher air humidity, greater concentrations of negative air ions [4], and significantly lower noise levels, all of which may positively influence immune function. Studies have shown that short-term forest bathing can enhance natural killer (NK) cell activity and reduce pro-inflammatory cytokines such as interleukin-6 (IL-6) and tumor necrosis factor α (TNF-α) [5,6]. It can also increase the expression of cancer-related immune proteins such as perforin and granzyme B [7]. These effects are thought to result from both direct biochemical pathways involving plant-emitted phytoncides and indirect psycho-neuro-immunological mechanisms, such as cortisol regulation [8]. However, these benefits are typically transient and tend to wane within weeks after individuals leave the forest environment [9]. This underscores the urgent need to explore whether sustained forest exposure can yield cumulative effects, potentially modulating immunosenescence over time.

However, human studies face substantial methodological challenges, including individual variability, differences in physical activity patterns, and dietary habits, which complicate control over confounding variables. While epidemiological studies have documented statistical links between green spaces and health outcomes, these findings require experimental validation to establish causality [10]. Although short-term forest exposure boosts immune parameters (e.g., NK cell activity), whether these effects accumulate over time to delay immunosenescence remains unknown, as human studies cannot control for long-term confounders (e.g., diet, exercise).

In contrast, animal models offer a valuable alternative for long-term environmental studies due to their controlled variables, standardized genetic backgrounds, and consistent lifestyles. Recent research using aging mouse models has shown that continuous exposure to forest environments can confer cumulative health benefits [11]. Nevertheless, the specific effects of long-term forest exposure on immunosenescence remain unclear. In this study, we employed a D-galactose-induced aging mouse model to compare the impacts of long-term exposure to urban versus forest environments on immunosenescence. This research not only provides empirical evidence for environment–immune system interactions but also offers data-driven support for urban planners aiming to design green urban environments that promote healthy aging.

## 2. Materials and Methods

### 2.1. Experimental Materials

Forty-eight healthy male ICR mice, 8 weeks old, with an average body weight of 40.5 ± 2.4 g, were purchased from Chengdu Dashuo Laboratory Animal Co., Ltd., Chengdu, China. The main reagents and instruments used in the experiment are listed in Table 1.

### 2.2. Study Sites

**Figure 1:**
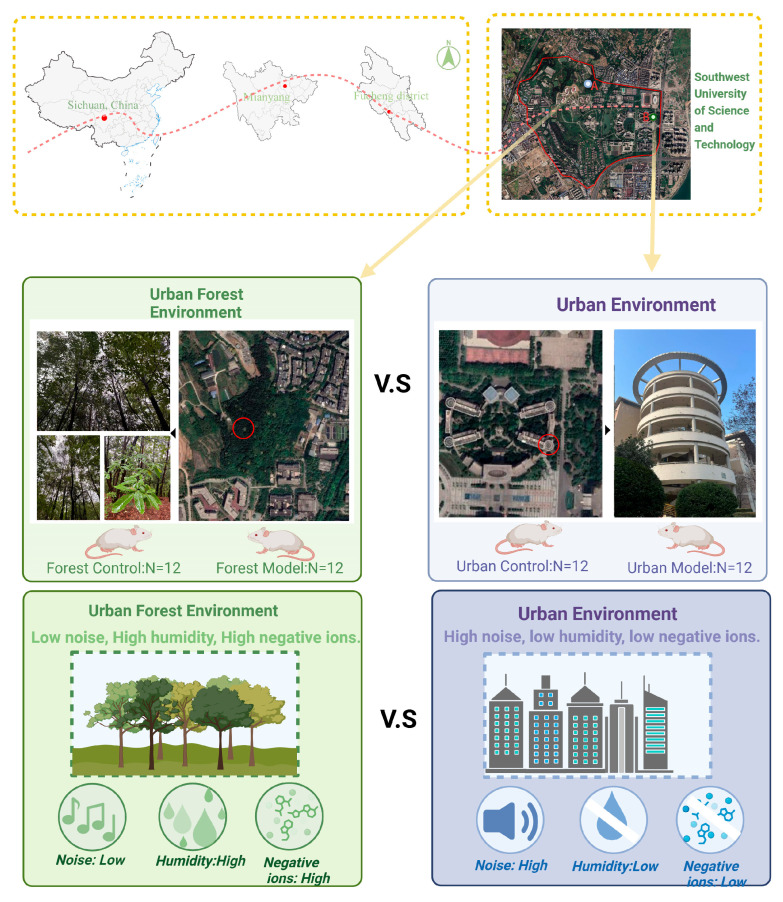
The experimental sites of this study.

As shown in Figure 1, this study selected typical urban and urban forest environments as the experimental locations. The typical urban environment is characterized by buildings, low vegetation coverage, and proximity to internal transportation routes and external traffic highways. The urban forest environment is primarily composed of forests with no buildings, high vegetation coverage, and tree species such as Ailanthus, Chinese fir, and Masson pine, along with forest paths. The straight-line distance between the two experimental locations is approximately 2 km. Animal experiments were conducted in areas with minimal human activity in both locations to minimize the impact of human presence on the mice.

### 2.3. Procedure

The experiment was approved by the Ethics Committee of Southwest University of Science and Technology (approval number: L2024014). The experimental mice were initially acclimated in an SPF-grade animal facility for one week to eliminate transport stress and allow them to adjust to the new environment. After the acclimation period, 48 mice were randomly selected and divided into four groups with 12 mice in each group: urban control group (UC), forest control group (FC), urban model group (UM), and forest model group (FM). Mice were housed in large cages, with each group in a separate cage.

The UC and UM groups were housed in the urban environment, while the FC and FM groups were housed in the forest environment. Mice in the UC and FC groups received daily subcutaneous injections of 0.1 mL/kg body weight of saline, while mice in the UM and FM groups received daily subcutaneous injections of 0.1 mL/kg body weight of D-galactose solution at a concentration of 0.020 g/mL [12]. Injections were administered for 56 consecutive days. Throughout the experiment, mice had free access to food and water, and bedding was changed every three days. Mice body weight was measured weekly, and the injection dose was adjusted based on the body weight.

### 2.4. Environmental Monitoring

During the experiment, environmental parameters such as temperature, humidity, negative ion concentration, and ambient noise were recorded simultaneously by two independent teams at both study sites during each monitoring session (at 8:00 a.m., 12:00 p.m., 4:00 p.m., and 8:00 p.m. daily). Measurements were taken at 3 s intervals for 3 consecutive recordings, with repeat measurements taken every 2 min, for a total of 6 recordings at each time point. Daily averages were calculated for statistical analysis.

### 2.5. Lymphocyte Cells Analysis

**Figure 2:**
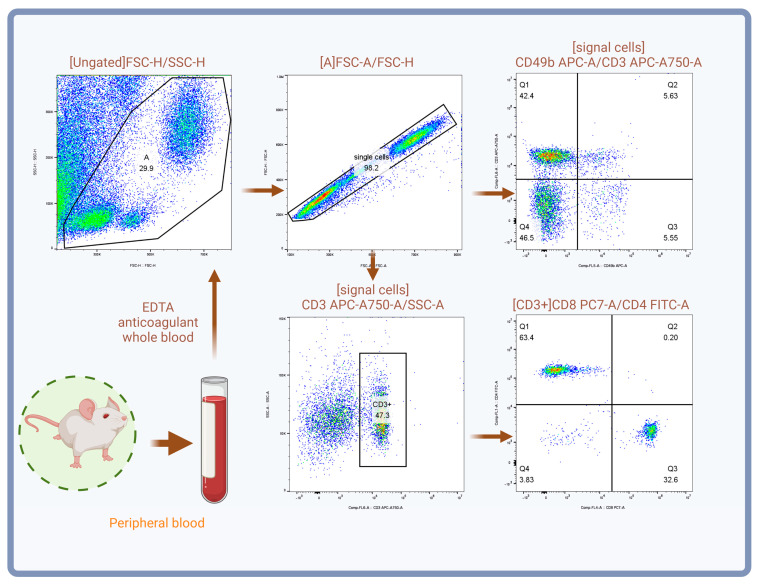
Diagram of flow cytometry detection process.

On the day of the final injection, mice were fasted overnight. The following morning, mice were weighed, and six mice from each group were randomly selected. After anesthesia with ether, blood was collected by ocular puncture and placed in EDTA anticoagulant tubes. The tubes were gently rolled twice on the tabletop to ensure proper mixing of the blood and anticoagulant. A 100 µL sample from each blood collection was transferred into flow cytometry tubes, followed by the addition of 1 µL of CD3, CD4, CD8, and CD49b antibodies. The samples were incubated at 4 °C for 30 min. After incubation, 2 mL of 1× ACK Lysis Working Buffer (diluted from 10× ACK Lysis Buffer) was added, and hemolysis was performed on ice for 10 min. The samples were centrifuged at 300× *g* for 5 min, and the supernatant was discarded. The cell pellet was resuspended in 2 mL PBS, centrifuged again at 300× *g* for 5 min, and the supernatant was discarded. The final cell pellet was resuspended in 400 µL PBS and immediately analyzed by flow cytometry. Flow cytometry data were analyzed using a hierarchical gating strategy. Briefly, debris was first excluded by FSC-H/SSC-H, followed by single-cell selection via FSC-A/FSC-H; within single cells, NKT cells (CD3^+^CD49b^+^) and NK cells (CD3^−^CD49b^+^) were identified based on CD3 and CD49b expression, with subsequent analysis of CD4^+^ and CD8^+^ subsets within the CD3^+^ population. The procedure is outlined in Figure 2.

### 2.6. Immune Organ Index Measurement

After blood collection, mice were humanely euthanized, and the thymus and spleen were carefully dissected and separated from connective tissue. The organs were washed in physiological saline and excess water was removed with absorbent paper. The organ weight was measured using an analytical balance (precision: 0.0001 g). The organ index was calculated as the organ weight (mg) divided by the body weight of the mouse (g).

### 2.7. Immune Factor Analysis

On the same day as blood sampling, the remaining six mice from each group were anesthetized with ether. Blood was collected via ocular puncture into 1.5 mL clean centrifuge tubes. After standing for an appropriate period, the blood was centrifuged at 3000 rpm for 10 min at 4 °C using a refrigerated centrifuge (5804R, Eppendorf, Hamburg, Germany). The supernatant was collected and used for immune factor (IL-2, IL-6, TNF-α, IFN-γ) level detection using ELISA kits.

### 2.8. Data Analysis

Experimental data were analyzed using SPSS 28.0 software, and one-way analysis of variance (ANOVA) was performed. A significance level of α = 0.05 was considered, with *p* < 0.05 indicating statistically significant differences between groups. Results are presented as means ± standard deviation (X ± S), and GraphPad Prism 9.0 was used for graphing the data.

## 3. Results

### 3.1. Microclimate

**Figure 3:**
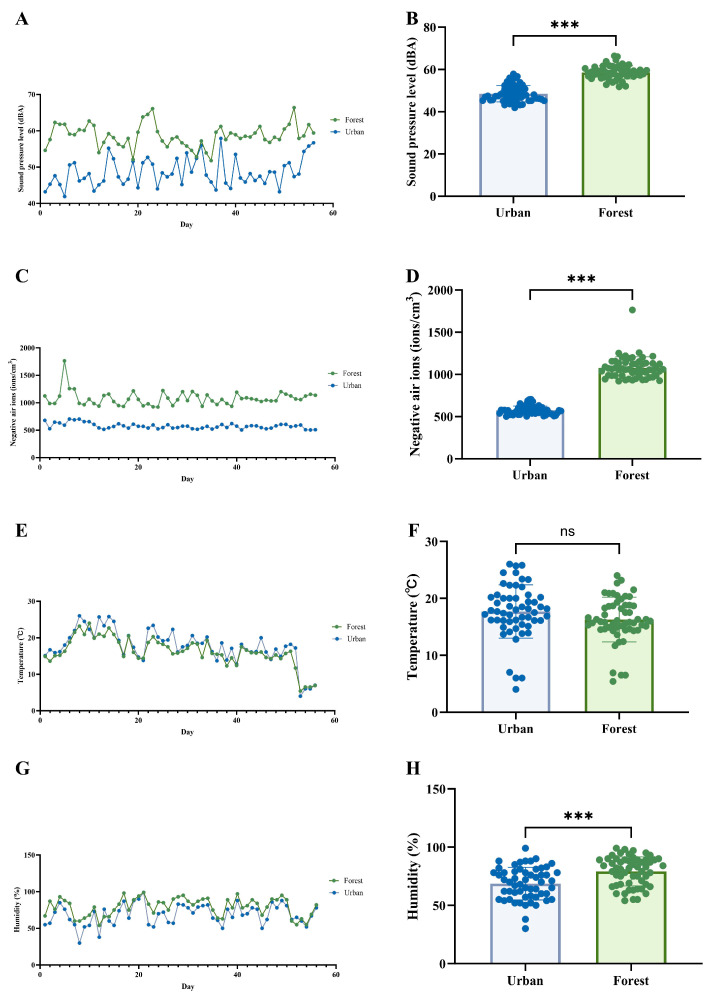
The microclimate at two experimental sites. (**A**,**B**) Daily sound pressure level records (**A**) and *t*-test comparison (**B**) between sites. (**C**,**D**) Daily negative ion concentration records (**C**) and *t*-test comparison (**D**). (**E**,**F**) Daily temperature records (**E**) and *t*-test comparison (**F**). (**G**,**H**) Daily humidity records (**G**) and *t*-test comparison (**H**). *** *p* < 0.001, and ^ns^ *p* > 0.05.

Figure 3 compares microclimatic factors between urban and forest environments, showing statistically significant differences (*t*-test, *p* < 0.05) in sound pressure levels, negative ion concentration, and humidity. Although temperature variations were observed between the two environments, these differences were not statistically significant. The summary of microclimate factors can be found in Table S1.

### 3.2. Weight

**Figure 4:**
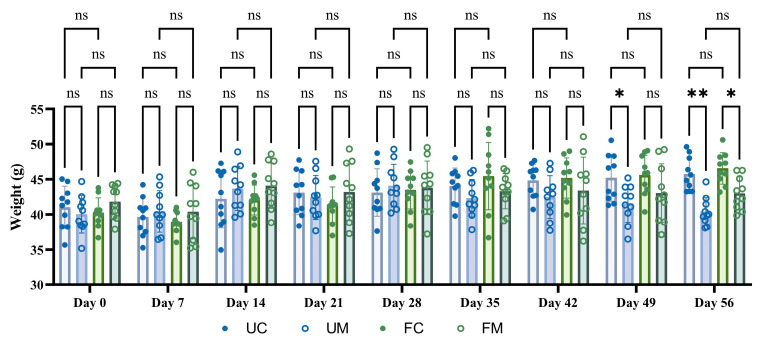
The weight of mice at two experimental sites. * *p* < 0.05, ** *p* < 0.01 and ^ns^ *p* > 0.05.

Figure 4 demonstrates no statistically significant differences in body weights across groups during the first four weeks (*p* > 0.05). From weeks 5–6, model group mice exhibited lower average body weights than their respective environmental controls, though these differences were not statistically significant. By week 7, the forest model group showed significantly reduced body weight compared to the forest control group (*p* < 0.05), while no significant difference emerged between forest and urban model groups. This weight reduction pattern became more pronounced in week 8, with model groups displaying significantly lower body weights than their matched environmental controls (*p* < 0.05).

### 3.3. Immune Organ Index

**Figure 5:**
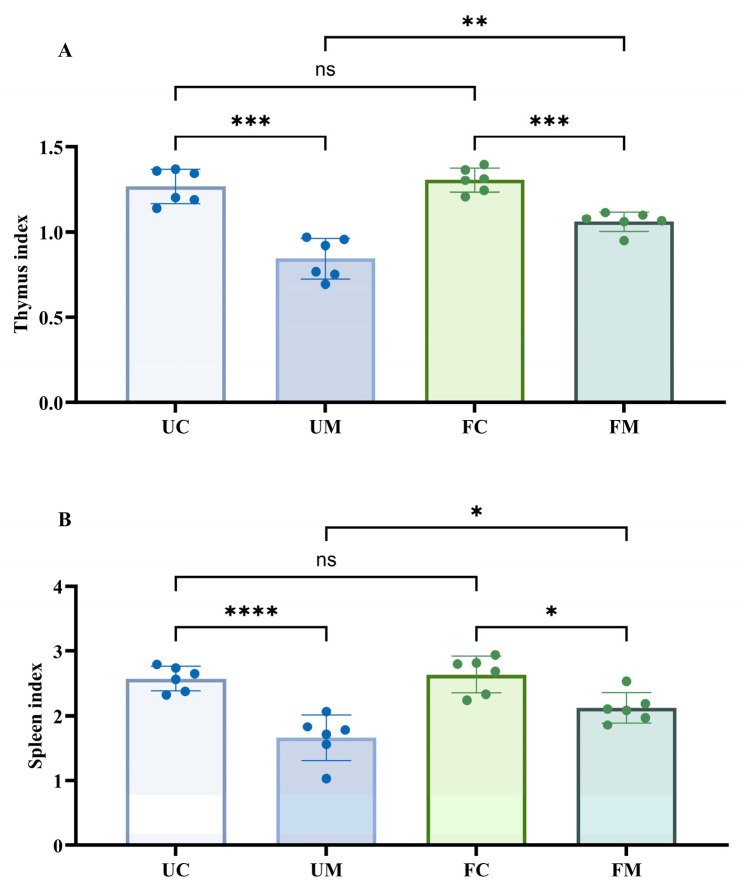
The immune organ index of mice at two experimental sites. (**A**) The thymus index. (**B**) The spleen index. * *p* < 0.05, ** *p* < 0.01, *** *p* < 0.001, **** *p* < 0.0001, and ^ns^ *p* > 0.05.

Figure 5 presents the immune organ index analysis by one-way ANOVA. Compared to the urban and forest control groups (UC/FC), both model groups (UM/FM) showed significantly reduced thymus and spleen indices (*p* < 0.05). While no significant differences were observed between the FC and UC groups, the FM group demonstrated significantly higher organ indices than the UM group (*p* < 0.05).

### 3.4. T-Lymphocyte Subpopulations

**Figure 6:**
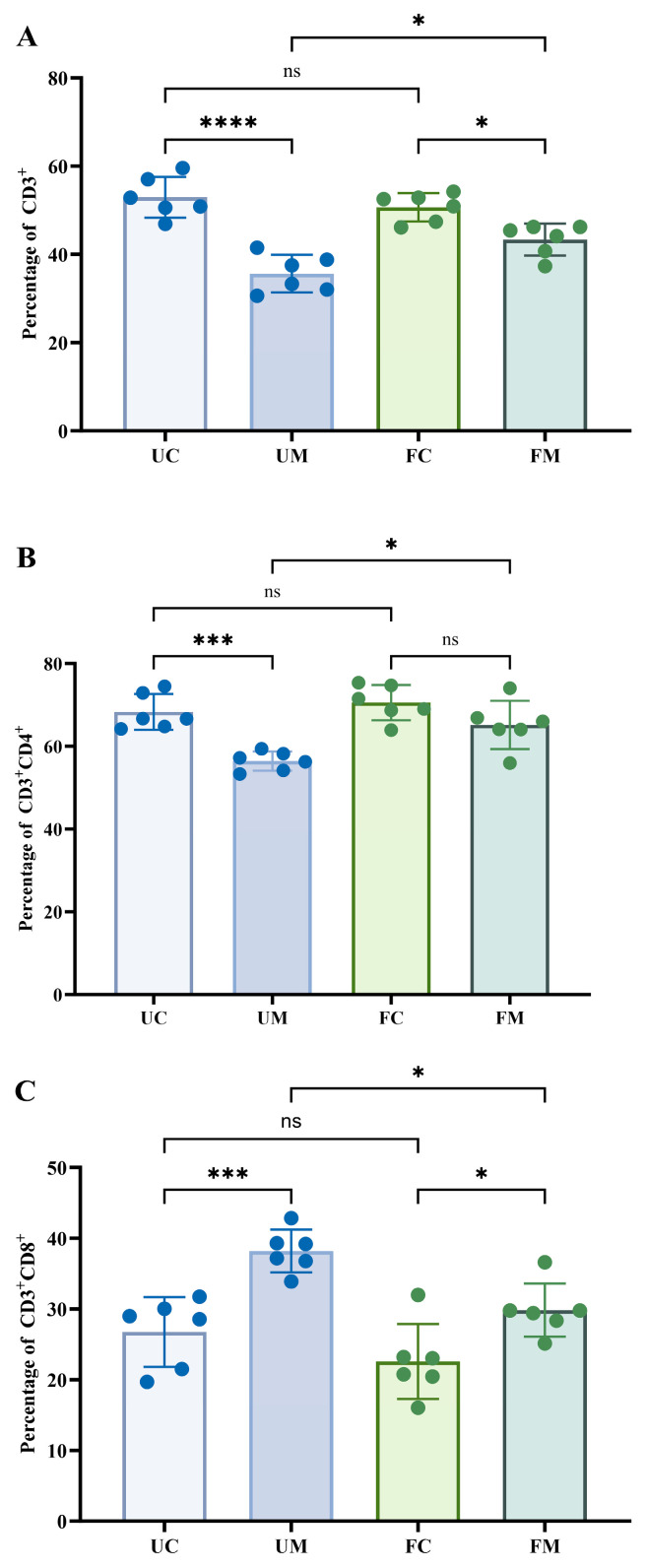
T lymphocyte subsets in mouse serum at two experimental sites. (**A**) The percentage of CD3^+^. (**B**) The percentage of CD3^+^CD4^+^. (**C**) The percentage of CD3^+^CD8^+^. * *p* < 0.05, *** *p* < 0.001, **** *p* < 0.0001, and ^ns^ *p* > 0.05.

Figure 6 displays the changes of T-lymphocyte subpopulations in peripheral blood across experimental groups. Significant differences emerged between model groups (UM/FM) and their respective controls (UC/FC) for all measured parameters, except for the CD3^+^CD4^+^ ratio between FM and FC groups (*p* > 0.05). Notably, the FM group exhibited significantly higher proportions of both CD3^+^ cells and CD3^+^CD4^+^ cells, along with significantly lower proportions of CD3^+^CD8^+^ cells, compared to the UM group (*p* < 0.05).

### 3.5. NK and NKT Cells

**Figure 7:**
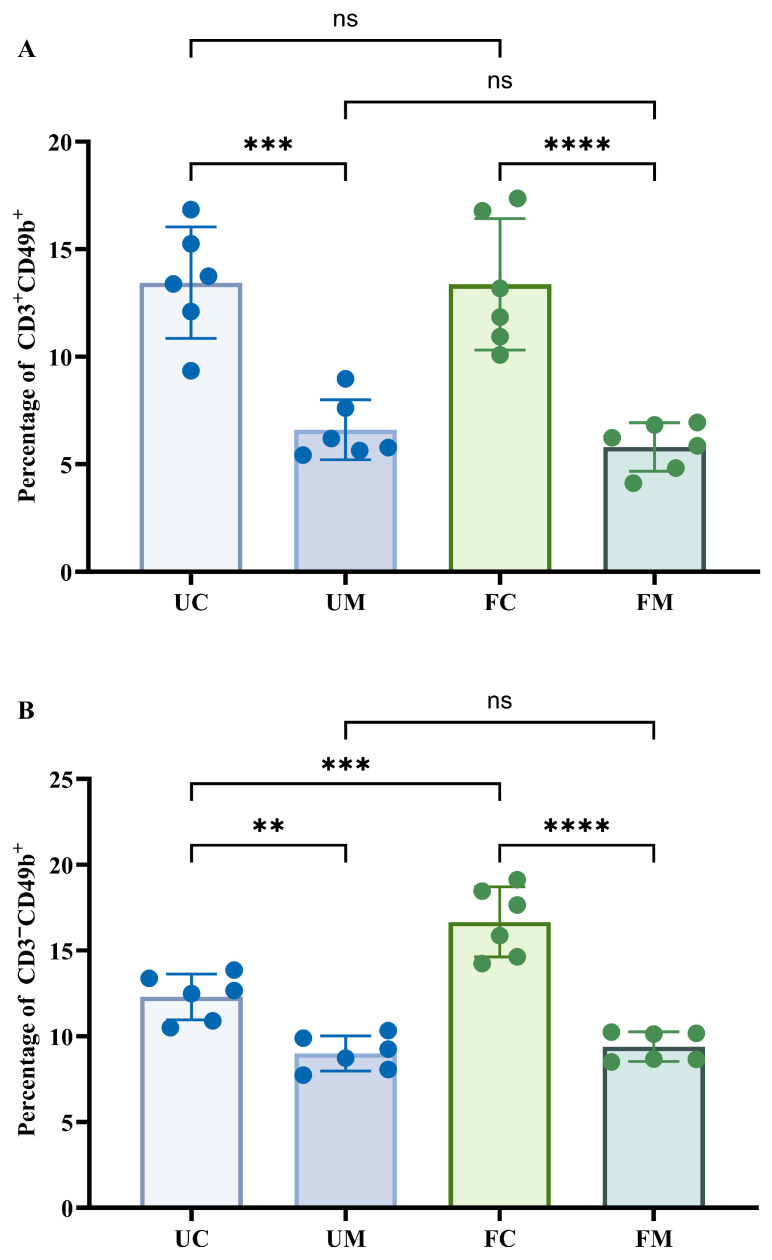
NK cells and NKT cells in mouse serum at two experimental sites. (**A**) The percentage of CD3^+^CD49b^+^. (**B**) The percentage of CD3^−^CD49b^+^. ** *p* < 0.01, *** *p* < 0.001, **** *p* < 0.0001, and ^ns^ *p* > 0.05.

Figure 7 presents the proportions of NK (CD3^−^CD49b^+^) and NKT (CD3^+^CD49b^+^) cells in peripheral blood. Compared to control groups (UC/FC), both model groups (UM/FM) showed significantly reduced proportions of NK and NKT cells (*p* < 0.05). While no significant differences emerged between FM and UM groups, the FC group exhibited a significantly higher NK cell proportion than UC (*p* < 0.05), despite comparable NKT cell levels between these controls.

### 3.6. Immune Factors

**Figure 8:**
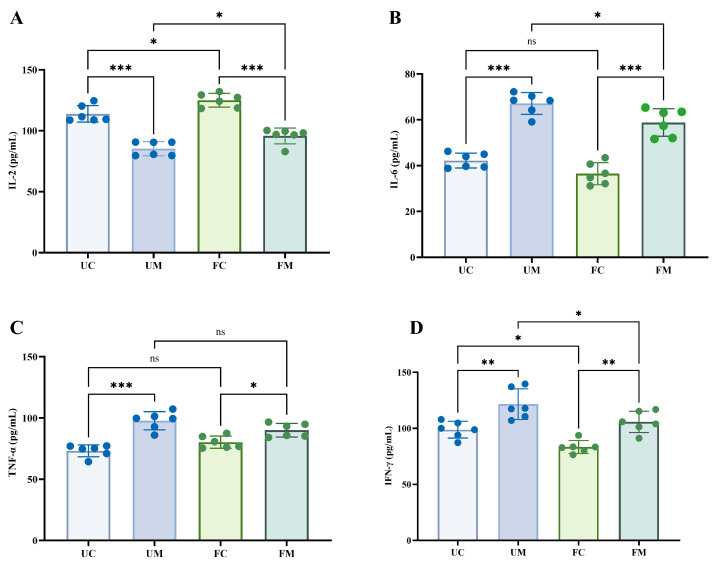
Immune cytokines in mouse serum at two experimental sites. (**A**–**D**) The serum cytokine concentration: (**A**) IL-2, (**B**) IL-6, (**C**) TNF-α, and (**D**) IFN-γ. * *p* < 0.05, ** *p* < 0.01, *** *p* < 0.001, and ^ns^ *p* > 0.05.

Figure 8 reveals cytokine differences across experimental groups. While serum interleukin-2 (IL-2) concentrations were significantly lower in the UM and FM groups compared to UC and FC controls (*p* < 0.05), the opposite pattern was observed for IL-6, TNF-α, and interferon γ (IFN-γ), with significantly higher levels in model groups (*p* < 0.05). The FC group exhibited elevated IL-2 but reduced IFN-γ concentrations relative to UC (*p* < 0.05), with comparable IL-6 and TNF-α levels. Notably, FM showed higher IL-2 but lower IL-6 and IFN-γ concentrations versus UM (*p* < 0.05). The intergroup comparisons of Weight, Immune Organ Index, T-Lymphocyte Subpopulations, NK and NKT Cells, and Immune Factors are summarized in Table S2.

## 4. Discussion

### 4.1. Key Findings

This study, using a D-galactose-induced aging mouse model, is the first to compare the effects of long-term exposure to urban and forest environments on immunosenescence. The results showed that the forest environment significantly alleviated age-related thymic atrophy, T lymphocyte dysfunction, and cytokine dysregulation. The findings are consistent with recent studies suggesting that urban green spaces mitigate immune stress responses caused by pollution [13], thus providing experimental evidence for the theoretical framework of “forest medicine” and supporting the consideration of urban forests as an environmental intervention to improve immune health in aging populations.

### 4.2. Potential Mechanisms of Forest Environment on Immunosenescence

Both the urban and forest model groups exhibited typical features of immunosenescence [14], confirming the successful establishment of the aging model. The smaller degree of change in aging-related indicators in the forest group suggests that one of the protective mechanisms of forest environments may lie in preserving the structural integrity of immune organs. Thymic atrophy during aging affects naïve T cell output, which in turn impairs immune function [15]. As aging progresses, the proportions of T cells (CD3^+^), helper T cells (CD3^+^CD4^+^), and cytotoxic T cells (CD3^+^CD8^+^) shift significantly [16,17]. Our findings revealed age-consistent changes in T, helper T, and cytotoxic T cell populations in both groups. Furthermore, decreases in serum levels of NKT cells (CD3^+^CD49b^+^), which play a critical role in immune tolerance and autoimmunity regulation and NK cells (CD3^−^CD49b^+^), key components of innate immunity, suggest impaired immunosurveillance, potentially increasing susceptibility to autoimmune diseases. These lymphocyte alterations reflect histopathological changes in immune organs and may represent a key contributor to increased susceptibility caused by age-related immunosenescence [18]. This degenerative process is characterized by lymphocyte depletion coupled with elevated pro-inflammatory cytokines, consistent with inflammaging [19].

An essential mechanism by which immune cells function involves the secretion of cytokines such as IL-2, IL-6, TNF-α, and IFN-γ [20,21,22]. In our study, the model group showed decreased IL-2 and elevated IFN-γ levels, indicating reduced immune surveillance and a potential decline in the immune system’s ability to clear infections, tumor cells, and repair aged tissues. The increased levels of TNF-α and IL-6 may reflect immune dysregulation and excessive immune cell activation, thereby elevating the risk of immune-related diseases. These cytokine alterations are closely associated with a range of age-related chronic diseases [23]. Except for TNF-α, the forest group exhibited milder changes in immune cytokines compared to the urban group, suggesting a slower progression of immune aging in mice residing in forest environments. Together with the changes in immune cells, these results further support the immune-protective effects of forests. Notably, even in young control mice, IL-2 and IFN-γ levels differed significantly between the forest and urban groups, indicating that long-term residence in forest environments can also enhance immune function in younger individuals. This aligns with recent reviews suggesting that forest exposure benefits not only older but also younger populations [24].

Ross and Jones [25] proposed that the immune-promoting effects of forests are largely attributable to beneficial compounds such as phytoncides and developed virtual reality-based forest therapy protocols to simulate forest immersion for patients unable to access outdoor environments. Forests may delay immunosenescence by improving air quality and reducing environmental stressors, which collectively suppress chronic inflammation [11]. Forests are rich in negative air ions, which have been shown to suppress inflammation mediated by leukocyte infiltration [26], and volatile organic compounds such as α-pinene can enhance NK cell activity [9]. Natural forest sounds have also been shown to alter salivary cortisol levels [27].

### 4.3. Implications for Human Health and Urban Planning

Although this study is based on a rodent model, its findings have significant public health implications for understanding the potential role of urban forest interventions in promoting healthy aging. Environmental stressors closely linked to immune decline, such as air pollution and noise, are markedly reduced in forest settings [28], confirming their stress-buffering properties. While murine and human immune systems differ, mice remain the preferred model due to conserved immune pathways and genetic tractability [29]. The observed alleviation of immunosenescence aligns with documented physiological benefits in forest-exposed humans [5,6,30], reinforcing translational relevance.

Most existing studies focus on short-term forest exposure. For example, three-day forest experiences can improve physiological indicators, yet these benefits diminish within seven days after leaving the forest [9]. Our long-term animal experiment uniquely demonstrates sustained immune modulation, bridging a critical research gap. This supports human observational findings: green spaces benefit middle-aged/older adults [31], improve cholesterol levels [32], enhance lung function [33], and reduce cardiovascular mortality [34]. While these studies show correlations, our controlled experiment strengthens causal inference [10].

Current green space standards largely emphasize spatial equity and quantitative metrics, as seen in the World Health Organization’s recommendation for 0.5 hectares standard within 300 m [35] and the U.S. Green Building Council’s LEED-ND’s 10% vegetated space requirement [36]. Our study suggests these standards should incorporate immunoregulatory functions through, first, prioritizing forest patches of adequate scale around aging communities to ensure stable negative ion concentrations; second, increasing tree canopy density for prolonged exposure; third, reconceptualizing urban forests as preventive healthcare infrastructure. This structural approach contrasts with short-term use paradigms, enabling immune system reconstruction through continuous natural exposure.

### 4.4. Limitations and Future Directions

This study has several limitations that warrant further investigation. First, although the D-galactose-induced aging mouse model is widely used in human aging research, its mechanism of accelerated aging differs somewhat from natural aging processes [37]. Future studies could employ naturally aged animal models or comparative studies across multiple models to assess the true effects of forest exposure more comprehensively on immunosenescence. Second, our study lacks empirical support from human populations. Prospective cohort studies involving older adults could be designed to track changes in immune function before and after relocating to forest-based communities, thereby testing the external validity of animal findings. Third, the exclusive use of male murine models, while controlling for sex-based variability, limits generalizability to female populations; future work should address potential sex-related differences in environmental immunomodulation. Lastly, as a complex natural exposure unit, the specific health-promoting mechanisms of forest environments remain to be elucidated. Future research should aim to isolate and identify key contributing factors (e.g., phytoncides, negative air ions, soundscape characteristics), explicitly test mechanistic pathways through phytoncide exposure assays and cortisol monitoring, and explore urban–forest transects with greater spatial separation to assess distance-dependent immunomodulatory effects.

## 5. Conclusions

This study systematically evaluated the effects of long-term exposure to urban forest environments on immune system aging using a murine senescence model. The results demonstrate that compared to general urban environments, urban forest habitats exhibit distinctive microclimatic characteristics and significantly attenuate age-related degeneration of immune organs and aberrant expression of inflammatory cytokines as well as alterations in lymphocyte subsets in mice. This study offers important implications for enhancing immune health among aging urban populations. Future urban planning should extend beyond merely considering green space accessibility and recreational functions, placing greater emphasis on the immunomodulatory and healthy-aging potentials of forest-type green spaces, thereby achieving synergistic development between environmental quality improvement and public health promotion.

## Figures and Tables

**Table 1:** Major reagents and instruments used in this study.

Name	Catalog Number/Model	Manufacturer
Rat Anti-Mouse CD8α-PE/CY7 (53-6.7)	1550-17	SouthernBiotech, Birmingham, AL, USA
APC-Cy™7 Hamster Anti-Mouse CD3e	557, 596	BD Pharmingen™, Franklin Lakes, NJ, USA
FITC anti-mouse CD4 Antibody	100, 406	Biolegend, San Diego, CA, USA
APC Anti-Mouse CD49b Antibody [DX5]	E-AB-F1116E	Elabscience, Wuhan, China
Lysing Buffer	555, 899	BD Pharmingen™, Franklin Lakes, NJ, USA
10× ACK Lysis Buffer	E-CK-A105	Elabscience, Wuhan, China
Mouse interleukin 2 (IL-2) ELISA kit	MM-0701M1	Meimian, Yancheng, China
Mouse interleukin 6 (IL-6) ELISA kit	MM-0163M2	Meimian, Yancheng, China
Mouse tumor necrosis factor α (TNF-α) ELISA kit	MM-0132M2	Meimian, Yancheng, China
Mouse interferon gamma (IFN-γ) ELISA kit	MM-0182M1	Meimian, Yancheng, China
Flow cytometer	CytoFlex	Beckman, Brea, CA, USA
Refrigerated centrifuge	Microfuge 20R	Beckman, Brea, CA USA
Microplate Reader	SH-1000Lab	Hitachi High, Tokyo, Japan

## Data Availability

All the data in this study are included in the article.

## Supplementary Materials

The following supporting information can be downloaded at: https://www.mdpi.com/article/10.3390/biology14080998/s1, Table S1: Comparative analysis of environmental parameters between urban and forest sites; Table S2: Summary of inter-group comparisons for physiological and immunological biomarkers.
