# Supplementary material for: Effects of Forest Environments in Attenuating D-Galactose-Induced Immunosenescence: Insights from a Murine Model
Source: Biology (Basel). 2025 Aug 5;14(8):998. doi: 10.3390/biology14080998 (PMC12383387; doi:10.3390/biology14080998)
Supplement: Supplementary file 1 [file biology-14-00998-s001.zip › biology-3740809-supplementary.pdf]

Table S1. Comparative analysis of environmental parameters between urban and forest sites

Parameter	Urban	Forest	P-value	Summary
SPL (dBA)	48.25 ± 3.88	58.59 ± 3.12	<0.001	***
Negative air ions (ions/cm^3)	578.13 ± 50.58	1074.97 ± 131.13	<0.001	***
Temperature (°C)	18.11 ± 4.68	16.63 ± 3.92	0.08	ns
Humidity (%)	68.54 ± 13.99	79.28 ± 12.41	<0.001	***

Note: Data presented as mean ± standard deviation (SD). Statistical comparisons by Student's t-test.

Table S2. Summary of inter-group comparisons for physiological and immunological biomarkers

Parameter	Results				P-value			
	UC	FC	UM	FM	UC vs. UM	UC vs. FC	UM vs. FM	FC vs. FM
Weight (g, day 0)	41.02±2.66	40.03±2.67	40.27±2.21	41.81±2.01	0.8304	0.854	0.3574	0.3995
Weight (g, day 7)	39.67±2.35	40.44±2.8	38.95±1.63	40.38±3.8	0.9579	0.9107	>0.9999	0.6339
Weight (g, day 14)	42.2±4.62	43.69±2.58	42.06±1.97	44.11±3.2	0.7728	0.9998	0.9948	0.3763
Weight (g, day 21)	43.12±3.23	42.42±3.54	41.38±2.12	43.22±4.23	0.9638	0.7255	0.956	0.7276
Weight (g, day 28)	43.12±3.41	44.11±2.74	43.52±2.75	43.82±4	0.8935	0.9942	0.998	0.9982
Weight (g, day 35)	44.06±2.4	42.32±2.44	45.47±4.77	43.31±2.58	0.5824	0.8752	0.5459	0.7314
Weight (g, day 42)	44.86±2.48	42.47±3.13	45.19±2.86	43.41±4.97	0.3239	0.9932	0.945	0.7841
Weight (g, day 49)	45.21±2.76	41.57±2.94	45.6±2.88	43.07±4.41	**0.0439**	0.9959	0.591	0.5277
Weight (g, day 56)	45.75±2.54	40.4±2.1	46.57±2.2	43.02±2.46	**0.002**	0.8636	0.0715	**0.047**
Spleen index	2.57±0.19	1.66±0.35	2.64±0.28	2.12±0.23	**<0.0001**	0.9781	**0.0377**	**0.0183**
Thymus index	1.27±0.1	0.84±0.12	1.31±0.07	1.06±0.06	**<0.001**	0.89	**0.003**	**<0.001**
$CD3^+$ (%)	52.97±4.62	35.64±4.28	50.68±3.22	43.35±3.59	**<0.0001**	0.7525	**0.0149**	**0.0215**
$CD3^+CD4^+$ (%)	68.28±4.34	56.43±2.35	70.53±4.27	65.16±5.83	**0.0007**	0.8106	**0.0122**	0.1789
$CD3^+CD8^+$ (%)	26.75±4.93	38.2±3.02	22.58±5.29	29.85±3.75	**0.001**	0.3678	**0.0161**	**0.0408**
$CD3^+CD49b^+$ (%)	13.45±2.59	6.61±1.4	13.37±3.05	5.8±1.13	**0.0002**	>0.9999	0.9197	**<0.0001**
$CD3^-CD49b^+$ (%)	12.29±1.34	9±1.02	16.66±2.05	9.39±0.86	**0.0029**	**0.0001**	0.9602	**<0.0001**
IL-2 (pg/mL)	113.93±6.8	85.31±5.83	125.04±5.67	95.76±6.48	**<0.001**	**0.03**	**0.04**	**<0.001**
IL-6 (pg/mL)	42.2±3.23	67.13±4.76	36.47±4.8	58.82±6.02	**<0.001**	0.2	**0.03**	**<0.001**
TNF-α (pg/mL)	73.21±4.92	97.76±7.37	80.23±5.01	89.97±5.55	**<0.001**	0.19	0.13	**0.04**
IFN-γ (pg/mL)	98.81±7.43	121.6±13.64	83.37±5.76	105.76±9.48	**0.003**	**0.05**	**0.04**	**0.003**

Note: Data presented as mean ± standard deviation (SD). Significant p-values are bolded ($p<0.05$, one-way ANOVA with Tukey's post-hoc test). Group abbreviations: UC (urban control), FC (forest control), UM (urban model), FM (forest model). Organ indices expressed as organ weight (mg) to body weight (g) ratio.
